# Experiences of pediatric cancer patients (age 12–18 years) with extensive germline sequencing for cancer predisposition: a qualitative study

**DOI:** 10.1038/s41431-024-01565-3

**Published:** 2024-02-27

**Authors:** Sebastian B. B. Bon, Roel H. P. Wouters, Jette J. Bakhuizen, Marjolijn C. J. Jongmans, Marry M. van den Heuvel-Eibrink, Martha A. Grootenhuis

**Affiliations:** 1 Princess Máxima Center for Pediatric Oncology, Utrecht, The Netherlands; 2 Department of Psychiatry, Amsterdam University Medical Center, University of Amsterdam, Amsterdam, The Netherlands; 3 Department of Genetics, University Medical Center Utrecht, Utrecht, The Netherlands; 4 Division of Child Health, UMCU-Wilhelmina’s Children’s Hospital, Utrecht, The Netherlands

**Keywords:** Human behaviour, Cancer genetics

## Abstract

This study explored the experiences and needs of adolescents, ranging from 12 to 18 years old, who have recently been diagnosed with cancer and participated in a nationwide germline genetic sequencing study within the context of pediatric oncology. The 21 adolescents in this qualitative interview study viewed genetic sequencing as an integral part of their cancer journey. They often characterized germline sequencing as “good-to-know” without specifying immediate utility. While the adolescents comprehended the significance of germline genetic sequencing, they were less focused on its potential long-term implications. Adolescents expressed a strong desire to be actively engaged in decisions related to genetics. They advocated for a participatory role in genetic decision-making from a young age onwards. They recommended that re-consent should be sought before re-analysis of their genetic data is performed and believe that patients should have the opportunity to provide (re-)consent once they reach adulthood. Moreover, the adolescents emphasized the importance of developing counseling materials that are not only concise but also visually attractive. In conclusion, this study underscores the positive perception that adolescents diagnosed with cancer hold regarding germline genetic sequencing. They articulate a strong interest in being actively involved in genetic decision-making. To address these articulated needs and preferences, we recommend the development of visually engaging counseling materials. These materials should effectively convey both the immediate and long-term implications of genetic sequencing, enabling adolescents with cancer to make informed decisions about genetic sequencing.

## Introduction

Sequencing of germline DNA is gradually being implemented as a routine practice in the care of children with cancer. Recent studies estimate that 10% of children with cancer have a genetic predisposition [1, 2]. Advances in sequencing technologies and the identification of a growing number of genetic predisposition syndromes have expanded the range of genes that can be tested, as well as the number of patients that can be tested. This means that it is technically possible to routinely sequence all children with cancer, rather than only sequencing patients with a high risk of carrying a specific predisposition. Furthermore, there is a tendency to perform germline sequencing at an earlier stage of the cancer trajectory, i.e., as part of the diagnostic process [3, 4].

Testing children for cancer predisposition is a topic of ongoing ethical debate [5]. This debate encompasses various issues, including what information should be provided during the consent process, what appropriate timing of consent would be, how children should be involved in decision-making, and whether re-consent is desirable when children reach the age of majority. With the introduction of germline genetic sequencing for all pediatric cancer patients, regardless of their anticipated risk of having a cancer predisposition, there is a need for more knowledge on how families, and especially adolescents, perceive and experience this routine sequencing. Previous studies that explored the experiences of children with cancer predisposition testing have primarily been conducted in families with highly penetrant predispositions or with a high risk of carrying a predisposition [6–8], in children who had not been diagnosed with cancer [9], and in children with a poor prognosis, mainly to identify somatic aberrations in the tumor [10–14]. Studies report several consequences of sequencing, with positive effects such as higher perceived personal control coexisting with negative effects such as psychosocial burden caused by testing itself and the disclosure of test results [8, 11, 12]. Still, it is largely unknown to what extent these outcomes apply to the wider pediatric oncology population, particularly in the context of adolescents with childhood cancer.

To elucidate families’ experiences with extensive germline sequencing as a routine part of pediatric oncology practice, we conducted the REFLECT study (Reactions and Emotions of Families Linked to Extensive sequencing in childhood Cancer patienTs). In this paper we will describe the results of the REFLECT-Interviews. Where we interviewed pediatric cancer patients (aged 12–18 years) who were awaiting test results of germline sequencing at the time of interview, approximately 6 months after their cancer diagnosis. These patients will further be referred to as adolescents or interviewees. We present the experiences of these adolescents by describing six emerging themes. Knowledge about the experiences of these adolescents is essential for an appropriate implementation of genetic sequencing in routine care.

## Materials and methods

### Context

Interviews were conducted with adolescents, diagnosed with cancer, participating in the PrediCT sequencing study [15]. In the PrediCT study a panel of 143 genes associated with childhood cancer was tested. The panel and the criteria that were used to select the genes can be found in Supplementary File 1. The sequencing study was offered to patients diagnosed at the Princess Máxima Center for Pediatric Oncology (age <19 years) between June 2020 and August 2022. Pathogenic or likely pathogenic variants in the panel were disclosed to patients and their parents (hereafter: families). For practical reasons, inclusion started and ended stepwise for patients with hematologic neoplasms, central nervous system tumors, and solid tumors. The PrediCT study did not include sequencing of tumor DNA.

Adolescents referred for evaluation by a clinical geneticist (based on their phenotypical characteristics) were not excluded from the sequencing study, but recruitment for the sequencing study was postponed until their evaluation had been completed.

From December 2021 onwards the REFLECT study, encompassing a questionnaire study for all family members (parents of children all ages; and patients aged 12–18) and an interview study (only patients aged 12–18) was offered in conjunction with the PrediCT study. Interviews took place between consent and disclosure of germline genetic sequencing results.

**Fig. 1:**
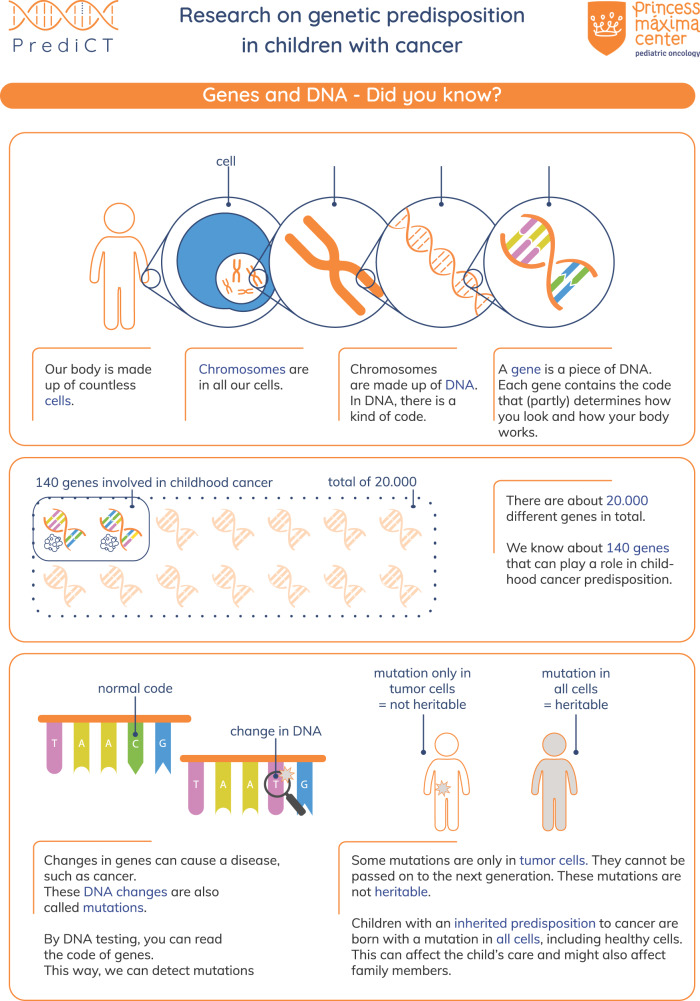
Infographic: genes and DNA - Did you know?. represents a translated version of the infographic used in the informed consent procedure for the PrediCT sequencing study.

Consent for sequencing was obtained about five months after cancer diagnosis by a physician-researcher. The informed consent procedure consisted of an introductory phone call and a more elaborate face-to-face information session supported by an information letter and an infographic, see Fig. 1. During this session, parents and adolescent patients were educated about topics such as genetic and hereditary causes of childhood cancer, the likelihood that a genetic explanation is found, and the potential implications of detecting a genetic cancer predisposition (including possibilities for early cancer detection and intervention, coping and living with knowledge about hereditary cancer risks and insurability issues). In children aged 12 to 15 years, both children and their parents or guardians were required to provide written consent. Upon reaching the age of 16, adolescents provided consent for sequencing by themselves.

### Interviews

**Fig. 2:**
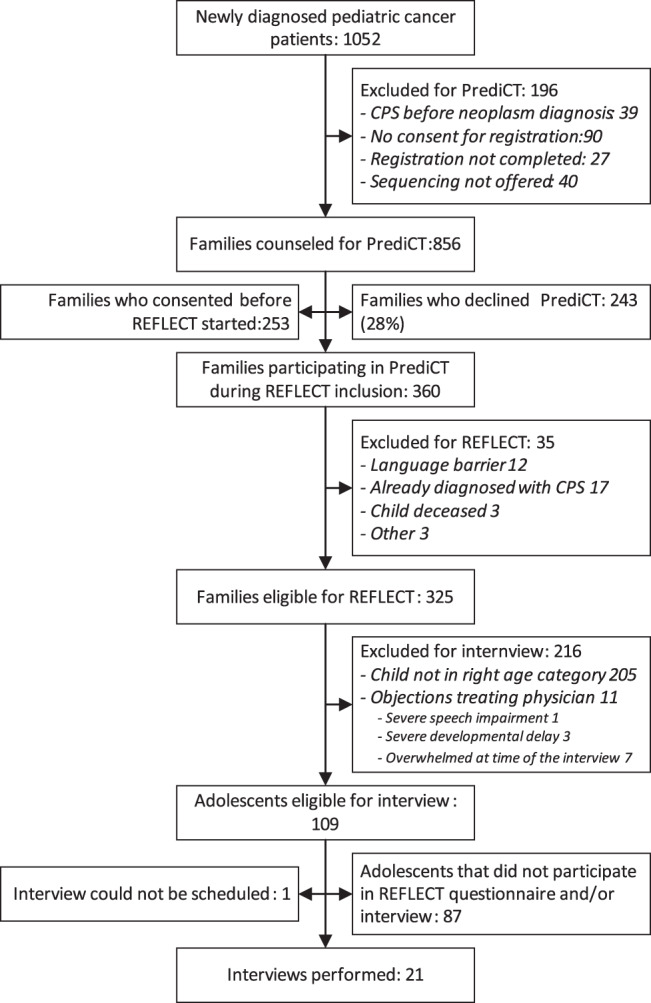
Study flowchart. This flowchart describes the recruitment, inclusion, and exclusion process of the interview study.

Participants of the PrediCT sequencing study between 12 and 18 years old were eligible for the REFLECT-Interview study, no purposive sampling was used. Exclusion criteria for the REFLECT-Interview study were: insufficient proficiency in Dutch, prior confirmation of a genetic cancer predisposition, or objection to an interview by their treating physicians (e.g., being overwhelmed by a cancer relapse or a stem cell transplant around the time of the interview). Recruitment for the interview study started in December 2021 and continued until December 2022. The inclusion for the study is visualized in Fig. 2. Main reasons for refusing participation in the interview study were the already high research burden and the burden of cancer treatment, and a lack of interest in this specific study.

Interviews were scheduled at the most convenient time and location for the adolescent: face-to-face at the hospital, face-to-face at home, or via a secured videoconferencing platform (Skype for Business). A semi-structured interview guide was used (Supplementary File 2). Topics include: adolescents’ motivation for participation in sequencing and perceived burdens, evaluation of the counseling process and provided material; views on future re-analysis (of genetic data or samples) and re-contacting (to share novel findings or insights), and views on obtaining re-consent for future use of data upon reaching the age of majority. One researcher (S.B.) conducted the interviews. Interviews lasted 20 min on average (range: 14–34 min) and were audio-recorded and transcribed verbatim.

### Data analysis

We adopted an inductive thematic approach to qualitatively analyze the interviews [16]. The first three interviews were coded independently by two authors (S.B. and R.W.) using NVivo (QSR International Pty Ltd. Version 12, 2018). Differences in coding were discussed until consensus was reached. Subsequent interviews were initially coded by one author (S.B.) and then systematically reviewed by a second author (R.W.). In an iterative process, interviews were recoded when new codes emerged [17]. The resulting code tree provided an overview of the topics and content. All authors then reviewed the code tree to identify emerging themes. Provisional themes were discussed until consensus was reached among the authors.

## Results

We conducted interviews with 21 adolescents who were awaiting results of germline genetic sequencing. Adolescents were aged 12 to 18, and of different disease groups, demographic information can be found in Table 1. We identified six emerging themes from the interviews, which will be described below.

**Table 1:** Interviewee and family characteristics.

Interviewee characteristics	*N* = 21
Gender	
Male	10 (48%)
Female	11 (52%)
Age	
Median age	15 years (range 12–18)
Mean age	15.3 years (SD 1.9)
Tumor type	
Hematological malignancy	7 (33%)
Solid tumor	8 (38%)
Central nervous system tumor	6 (29%)
Previously evaluated by a clinical geneticist^a^	
No	13 (62%)
Yes	8 (38%)
Treatment status at the time of the interview	
Active treatment	5 (24%) (Average time since diagnosis 6,8 months)
Follow-up care	16 (76%) (Average time since diagnosis 7,3 months)
Family characteristics	
Education level of the highest educated parent^b^	
Low	0
Middle	6
High	15
Biological parents	
Together	18
Not together	3
Birth country of parents	
The Netherlands	19
One parent outside The Netherlands	2

^a^Adolescents who were previously evaluated by a clinical geneticist could participate in an interview if a cancer predisposition was not identified by a clinical geneticist and they were now awaiting results of the sequencing study.

^b^Educational level defined according to Statistics Netherlands (CBS, Centraal Bureau voor de Statistiek), 2016: low educational level = no education, primary school, lower secondary education; middle educational level = upper secondary education, pre-university education, intermediate vocational education; high educational level = higher vocational education, university.

### Adolescents express casual attitudes towards sequencing

In general, interviewees displayed a casual attitude towards genetic sequencing, as if it was not a ’big deal’ to them. Interviewees stated that germline genetic sequencing was not a major event in their lives.

*“I have been through so many examinations that I did not perceive this to be something particularly huge or something that is more difficult or anything like that”-1*8yr, male

Furthermore, adolescents viewed genetic sequencing as an integral part of their cancer journey. They nuanced the possible impact of discovering a predisposition by pointing out that they already had cancer and therefore expected to be able to cope with learning about a predisposition as well. Additionally, they thought receiving sequencing results would only make them aware of their predisposition, since in that case the predisposition itself already exists.

*“To put it bluntly, this is how I felt about it from the beginning, like, it is what it is, and we just carry on. And if such a test then reveals it is actually within your family, well that would perhaps be interesting to know for later. But yeah, for me, to know that it ran in the family, well that would not make too much of a difference to me”-*18yr, male

Nearly half of the adolescents considered the probability of finding a predisposition to be low, in part because they had been told so by their healthcare providers. Most adolescents did not find it burdensome to wait for the results. A few interviewees acknowledged learning about genetic results could be somewhat challenging, but they were quick to add that the perceived benefits of knowing about cancer predisposition would outweigh any concerns. This confident attitude towards learning of sequencing results was widely shared among the interviewees.

Adolescents generally had not spoken with peers about sequencing of their DNA, even though other cancer-related issues were part of their conversations with friends or siblings.

*“No, we do have other things to talk about than genetic testing”-*17yr, female

Many interviewees mentioned the lack of additional burdens as a reason for agreeing to undergo sequencing, for example, no extra blood draws or hospital visits were required. In that sense, adolescents described germline sequencing as less burdensome than other studies or clinical procedures. Furthermore, almost all of them indicated that the genetic study was just one of many studies they participated in during their cancer trajectory.

*“It is just like, I do not need to do a blood draw for that, and I am quite good at blood draws anyway” -* 15 yr male

### Adolescents assert their own role in counselling and consent

Adolescents stressed the importance of making their own choices regarding genetic sequencing. Only one interviewee (15 years old) indicated it would have been acceptable if the parents had made the decision by themselves. The other adolescents emphasized that sequencing involved their life, their body, their DNA, and therefore they should have a decisive voice.

*“ Yes, because I feel that it’s my business. I also think that children who might be younger [than me] should be able to decide for themselves as well. So, if I want to know whether things are wrong or not, I believe I should have that certainty and thereby potentially prevent stress “*-16yr, female

“*I do think that it would be nice to ask about this. Yeah because, yeah it is my DNA, you know [laughs]. So, yeah, well, I cannot really explain it that well*”-13yr, male

One interviewee stated to appreciate the control over research participation, compared to the lack of control over the treatment trajectory.

Interviewees were specifically asked at what age children should be involved in decisions about sequencing. By and large, interviewees put the age threshold for (co-) deciding about genetic sequencing at ten years or below. They argued that deciding for yourself is important but believed younger children would not be able to comprehend genetics or that they would not be interested in the information.

*“[talking about a peer who is 11] I think she would be able to understand it rather well, even though I feel 11 is still a bit young. But anyway, I think from that age onward you are really able to understand, well, if I’ve had it, then my children will get it too, perhaps. I also feel that if you explain this in plain language that they would understand it, for sure, and also would be able to make that decision, actually”*-16yr, female

Most interviewees considered children from the age of 15 years to be capable of deciding independently on matters regarding genetic sequencing. Many adolescents felt they would have been ready to make such decisions at a younger age than their current age.

*“I think that if you are 12 or 13, you already know how it works and what the consequences could be for yourself later in life, potentially. So yeah, I would say about 13”-*16yr, female

*“I think perhaps even already from my age onwards. (…) Maybe you do not want to know it at all, you know, and so it seems wise to just be allowed to choose yourself”-*13yr, female

### Adolescents and parents engage in a joint decision-making process

In practice, the decision to participate in genetic sequencing was generally the outcome of a joint process between adolescents and their parent(s). This did not seem to be a contradiction with adolescent’s desire to make their own decision. Almost half of the interviewees (15–18 yr) stated they made the decision (mostly) on their own, half of the adolescents (12–18 yr) noted it was a joint decision. One interviewee felt barely involved in this process (12 yr) since his/her parents had a strong desire to participate. All other interviewees felt they had a significant role in the decision-making process and were satisfied with their role.

*“Even though I am allowed to decide for myself, I thought, well, it would be nice to talk about this with my parents for a bit, like, what do you think about this? But they also thought that I could participate, so then we filled out and sent in the forms”-*17yr, female

*“ Look, you know, I am not going to exclude my parents altogether, so it was just like: this is what it entails. Of course, it was my choice for sure “*17 yr, female

Some, mostly older, interviewees pointed out differences between their own attitudes regarding sequencing and those of their parent(s). In general, it seemed parents were more worried than their children, particularly about the implications of sequencing for future life insurance, increased risks for other family members, or future diseases. Adolescents did acknowledge these risks, but did not seem to worry as much, in line with the previously described casualness.

*“They [my parents] also wanted to hand-in their own DNA, like, do the same for us than we will also know. I said no, there is no need to, because, well for one reason or the other, they only need mine. So yes, they were slightly more concerned than I was [laughs]”-1*7yr, female

*“They are probably more chickened out about it than I am, but that was actually the case anyway, with everything, during my entire treatment (…) Just because they are parents”-1*3yr, male

### Adolescents appreciate genetic information as valuable

Adolescents considered genetic information to be valuable knowledge about themselves, even without specific thoughts about surveillance or preventative measures, they regarded it as “good to know”. Another frequently mentioned motivation for seeking genetic information was the possibility to identify risks for themselves, siblings, and other family members.

Most adolescents were mildly interested in finding the cause for their cancer; in general, they were more interested in consequences for the (near) future. One of these consequences concerned their own offspring, which was mentioned by half of the interviewees (regardless of age and gender).

*“I am kind of curious why this has happened to me, was it something I was prone for, something in the genes or stuff like that? So yeah, I was kind of curious about that. But wanting to know how this happened was not the number one reason for me”-1*7yr, female

Adolescents indicated that helping others, such as scientists or future families, was an important reason for participation in sequencing, although this motivation was seldom mentioned spontaneously. Many interviewees stressed they participated in several, if not all, studies offered to them.

*“Because we participate in virtually all research in this hospital. Just for science and stuff. Cause you guys are getting quite a lot out of the data, and well for us it is easy, just filling out something or whatever, and you guys can do a lot with that, that was kind of the reason”-1*8yr, male

### Adolescents prefer visually attractive and concise counseling materials

In general, interviewees were satisfied with the counseling they received about the sequencing study. Interviewees appreciated face-to-face counseling conversations with professionals. Many adolescents found the information letter lengthy and did not read all the information. Several interviewees pointed out, while they themselves did not have issues reading lengthy letters, other children may struggle with it. Interviewees found the infographic that was used during the counseling session to be more effective. Only two adolescents searched for additional information about genetics on the internet, while others felt they already had acquired enough knowledge about genetics in school. Three interviewees stressed counseling should be adjusted to better accommodate adolescents with cancer-related difficulties, such as fatigue or visual impairments.

When asked for suggestions to improve materials for future counseling, adolescents indicated a preference for concise and visual materials. Some interviewees mentioned videos as a preferred format, while others preferred a website or a paper-based infographic, like the one that was already provided.

### Adolescents want to play an active role in the future

Adolescents were successively presented with two hypothetical scenarios. The first scenario concerned whether children, who are too young to be involved in decision making regarding sequencing, should be asked for re-consent when they reach majority. Many of the interviewees expressed children should be asked for consent again for the storage and future re-use of their DNA when they become older. An overview of the reasons interviewees gave can be found in Table 2, illustrated by several quotes.

**Table 2:** Views on future reconsent.

Topic	Reason	Quote
Re- consent at majority	Patient might have forgotten about DNA, re-consent as a reminder	*“I think the child may have forgotten about that themself, that they put a signature under it, as it were. So, I guess that you have to send this [consent form] to them again”-*17yr, female
	Patient might have changed their opinion	*“Maybe you have changed at that point, also with regards to where you stand on these matters”-*17yr, male
	Patient might not have been able to (fully) decide for him/herself	*“I think that for each child you should perhaps ask that once again, when he is a bit older, when he is a bit more independent to think, like, what is stored in that hospital or somewhere else, do I still want that”-*13yr, female
	Because it is the right thing to do	*“Yes, that would be wise. And it would also be polite, I guess, to kind of ask again to a that child if this is actually okay”-*15yr, female
Re-consent for re-analysis	Because you need to be aware that testing is taking place	*“I think I would like to be asked beforehand. Uhm, yeah, because then I know that they are again doing something with my DNA again”-*12yr, female
	Patient might have changed their opinion	*“I think it would be nice to let this know. I believe that’s how I feel. At least, it would be fine to me, but I totally understand that it would be nice to know that. Or suppose someone does change his mind, that he decides he does not want this after all”-*17yr, male
	Because a different kind of genes could be tested	*“I would most likely say yes, but I do think it would be right to ask again whether I am okay with that, because it could possibly reveal something totally different. And this could include things that this person does not want to know at all. So yeah, I would ask”-*16yr, female

The second scenario involved the, again hypothetical, discovery of five new pediatric cancer genes, years after the participants’ initial sequencing results were obtained. Now, a scientist intends to reanalyze the DNA of all participants to investigate these newly discovered genes. The majority of adolescents felt they should be re-approached for consent before re-analysis of their DNA was conducted. An overview of the reasons they gave are displayed in Table 2.

Almost all adolescents advocated for an active future role, but they acknowledged that other individuals may have different preferences and perspectives on these matters. They often did this by emphasizing that their responses reflected their own opinions and that they did not want to decide for others regarding re-analysis and re-consent.

## Discussion

This study is one of the first to explore experiences of an unselected group of pediatric cancer patients with germline sequencing involving an extensive panel of genes implicated in cancer predisposition. This is an urgent topic given the increasing popularity of the idea that germline sequencing should routinely be offered to all pediatric cancer patients as part of their cancer trajectory [2–4]. The integration of germline sequencing into cancer trajectories of all pediatric cancer patients would be a new phase in the genomic era of pediatric oncology. It would mark a departure from decades of clinical practice when germline sequencing was only offered to a subset of patients with certain phenotypical characteristics such as their family history, tumor type, or morphological features [18]. The casual attitude of our interviewees suggest that germline sequencing proposed as a routine check-up offered to all, may be perceived differently by patients compared to when sequencing is offered to them because they are considered at-risk for a specific cancer predisposition that has been diagnosed in the family. It seems that in these families genetic sequencing is perceived as a more significant event in the lives of adolescents [6, 7], suggesting that the experiences with testing for a predisposition might be influenced by whether testing is pre-symptomatic or not. The experiences with sequencing in a routine setting also seem to differ from experiences with precision medicine programs in children with a hard-to-cure or rare cancer. In two previous studies into the experiences with such programs, hope of new treatment options and hope for improved chances of survival were reported as common motivations in parents and adolescents [10, 19]. In our interview study these hopes were not reported to be a motivation. Other reported motivations such as benefits for future patients and family members. Interestingly, interviewees who mentioned the significance for relatives typically referred to their siblings and not to (for example) uncles, aunts, or parents.

Our study underlines the importance and relevance of investing in strategies to involve pediatric cancer patients in counseling and consent for (germline) genetic sequencing. Patients interviewed in our study made clear that pediatric cancer patients want to play an active role in decision-making regarding the sequencing of their own DNA. Interviewees (who were between the ages of 12 and 18) thought that a right to be involved in such decisions should also apply to patients younger than themselves. These ideas are consistent with statements issued by genetics societies in the US and Europe, who emphasize that actively involving adolescents in such decisions should occur regardless whether individuals have reached the legal age of consent [20, 21]. Notably, interviewees pointed out that sequencing decisions were made in conjunction with their parents, and that they were fine with this being a joint process. This was also illustrated in an interview study with adolescents who participated in a biobank, in which adolescents also to varying degrees took a shared decision together with their parent(s) [22]. Hence, our interviewees did not seem to view the emphasis on their own autonomy and their wish (and reality) to make the actual decision in a dialog with their parents as contradictory. This finding resonates with previous research in empirical ethics pointing out that autonomy should be (and in fact is) regarded as having relational aspects and also that true autonomy is best operationalized by treating consent as a communicative process rather than a single contractual decision [23, 24]. The duality of claiming autonomy while acknowledging dependence may also be part of a coping strategy among pediatric cancer patients, which has previously been described [25]. Finally, it is worth noting that parents and adolescents might think different on decisions regarding genomics. Many studies suggest that parents have a less casual attitude towards sequencing, which may imply that within families there might be differences in perspectives between adolescents and their parents [26, 27]. As has been previously shown parents and adolescents might disagree on who should have the final say in decisions involving genomics, suggesting that conflicts could arise even though this was often not explicitly expressed by our interviewees [27, 28].

The challenges regarding informed consent for genetic testing of minors have been extensively discussed [29, 30]. One prominent issue concerns the age at which children are capable of making such decisions. It has been argued that adolescents possess many of the skills necessary to make informed medical decisions, although these skills are not yet fully developed [31, 32]. To optimally use adolescents’ decision-making capabilities, they require a supportive environment [31]. Healthcare professionals play a crucial role in creating such an environment by adapting their communication to adolescents’ emerging autonomy and adjusting the communication to their developmental stage [25, 33–35]. For example, by respecting and validating the adolescent as the patient and the one who makes the decisions, and by offering conversations without their parents. In practice this supportive environment is not always created [36]. In general adolescents might benefit from appropriately timing informed consent, with previous studies among parents suggesting that consent shortly after diagnosis is not optimal for all families [37]. Furthermore, our study highlights several aspects that could improve the supportiveness of the environment. The participants highly preferred the infographic over the information letter that was used, which is consistent with previous research into preferences of children [38, 39], but also with recent studies into parental experiences with precision medicine [40, 41]. Another aspect is drawing adolescents’ attention to long-term consequences of extensive germline sequencing such as surveillance, preventative measures and insurability based on the potential test results. These consequences were seldom spontaneously discussed by our interviewees. Interestingly, previous research has shown that parents do worry about such issues [42–44]. We suggest that next to technical information, such as what DNA is, information regarding the implications of sequencing should be given due consideration in counseling and in (visually attractive) counseling materials.

Given that interpretation and use of germline data may change over time leads to several ethical issues. One issue concerns whether DNA should be re-analyzed in case novel genes are associated with pediatric cancer and if patients should be recontacted in case re-analysis yields new insights [45, 46]. Most interviewees demanded to be informed before re-analysis would take place, stating that it should not be taken for granted that they would want to learn the results of re-analysis. Another issue is whether patients who participated in germline genetic sequencing when they were underaged (with the consent of their parents and/or consent of the minor) should (re-)consent to storage and utilization of their genomic data upon reaching majority [47]. Most of the interviewees indeed believe that children who did not provide consent themselves should provide consent when they reach majority, which is consistent with previous studies in both healthy adolescents and adolescents with health conditions [28, 48]. Consistent with our findings they mention reasons such as reconsent serving as a reminder and the importance of deciding for yourself. It is worth noting that the ideas of these adolescents do not align with current practices [47, 49]. While we acknowledge the existence of logistical and practical challenges, we believe it is crucial to explore strategies to navigate these challenges to give right to the autonomy and voices of children.

### Study limitations

Our study has several limitations. Firstly, it only included adolescents who agreed to participate in the genetic sequencing study. A substantial percentage of families choose not to pursue germline testing in a research setting, consistent with other studies [26, 44]. The perspectives and considerations of this group are an important topic for further research. Secondly, given that many adolescents decided not to participate in the interview study, there may be an inclusion bias towards interviewees who had an above-average interest in genetic sequencing, leading to a potential overrepresentation of adolescents who want to play a significant role in decisions regarding genetic sequencing. Thirdly, as the interviewees grew up in relatively well-educated families and since only a small minority had a parent born outside The Netherlands, we recommend that future studies try to include a more diverse population.

## Conclusions

The adolescents in our study express a positive attitude towards participating in a germline genetic sequencing study but also raise some important issues. It is apparent that adolescents believe that they have the right to play an active role regarding the use of their genetic information, both now and in the future. Given the increased usage of germline sequencing and its growing applications, it is crucial to stay engaged with the perspectives of young patients in this regard, as well as to adapt counseling methods to align with their needs.

### Supplementary information

Childhood cancer predisposition gene panel: PrediCT-study

Interview Guide

## Data Availability

The interview transcripts are not publicly available due to the sensitive and identifying nature of these data. However, we will provide a selection of relevant, unpublished short segments of interviews to others upon reasonable request.

## References

[1] Bakhuizen JJ, Hopman SMJ, Bosscha MI, Dommering CJ, Van Den Heuvel-Eibrink MM, Hol JA (2023). Assessment of Cancer Predisposition Syndromes In A National Cohort Of Children With a Neoplasm. JAMA Netw Open.

[2] Parsons DW, Roy A, Yang Y, Wang T, Scollon S, Bergstrom K (2016). Diagnostic yield of clinical tumor and germline whole-exome sequencing for children with solid tumors. JAMA Oncol.

[3] Oberg JA, Glade Bender JL, Sulis ML, Pendrick D, Sireci AN, Hsiao SJ (2016). Implementation of next generation sequencing into pediatric hematology-oncology practice: Moving beyond actionable alterations. Genome Med.

[4] Wise J (2019). Genome sequencing of children promises a new era in oncology. BMJ.

[5] Eichinger J, Elger BS, Koné I, Filges I, Shaw D, Zimmermann B (2021). The full spectrum of ethical issues in pediatric genome-wide sequencing: a systematic qualitative review. BMC Pediatr.

[6] Forbes Shepherd R, Werner-Lin A, Keogh LA, Delatycki MB, Forrest LE (2021). I need to know if I’m going to die young”: adolescent and young adult experiences of genetic testing for Li–Fraumeni syndrome. J Psychosoc Oncol.

[7] Alderfer MA, Lindell RB, Viadro CI, Zelley K, Valdez J, Mandrell B (2017). Should genetic testing be offered for children? The perspectives of adolescents and emerging adults in families with Li-Fraumeni syndrome. J Genet Couns.

[8] Weber E, Shuman C, Wasserman JD, Barrera M, Patenaude AF, Fung K (2019). A change in perspective”: exploring the experiences of adolescents with hereditary tumor predisposition. Pediatr Blood Cancer.

[9] Pervola J, Myers MF, McGowan ML, Prows CA (2019). Giving adolescents a voice: the types of genetic information adolescents choose to learn and why. Genet Med.

[10] Marron JM, DuBois SG, Bender JG, Kim AR, Crompton BD, Meyer SC (2016). Patient/parent perspectives on genomic tumor profiling of pediatric solid tumors: the Individualized Cancer Therapy (iCat) experience. Pediatr Blood Cancer.

[11] Hetherington K, Wakefield CE, Kunalan KPK, Donoghoe MW, McGill BC, Fardell JE (2022). Quality of Life (QoL) of children and adolescents participating in a precision medicine trial for high-risk childhood cancer. Cancers.

[12] McGill BC, Wakefield CE, Hetherington K, Munro LJ, Warby M, Lau L, et al. “Balancing Expectations with Actual Realities”: Conversations with Clinicians and Scientists in the First Year of a High-Risk Childhood Cancer Precision Medicine Trial. J Pers Med. 2020;10. 10.3390/jpm10010009. 10.3390/jpm10010009 PMC7151613 32075154

[13] Wakefield CE, Hetherington K, Robertson EG, Donoghoe MW, Hunter JD, Vetsch J (2023). Hopes, concerns, satisfaction and regret in a precision medicine trial for childhood cancer: a mixed-methods study of parent and patient perspectives. Br J Cancer.

[14] Sedig LK, Jacobs MF, Mody RJ, Le LQ, Bartnik NJ, Gornick MC, et al. Adolescent and parent perspectives on genomic sequencing to inform cancer care. Pediatr Blood Cancer. 2022;69. 10.1002/pbc.29791. 10.1002/pbc.29791 35735208

[15] Bakhuizen JJ. Dutch Trial Register, PrediCT - Predisposition to Childhood Tumors Tumors. 2020. https://onderzoekmetmensen.nl/en/trial/20548. Kassambara A. rstatix: pipe-friendly framework for basic statistical tests. 2020. https://rpkgs.datanovia.com/rstatix/.

[16] Braun V, Clarke V (2006). Using thematic analysis in psychology. Qual Res Psychol.

[17] Pope C (2000). Qualitative research in health care: Analysing qualitative data. Bmj.

[18] Reichman L, Goudie C. Recognition of cancer predisposition syndromes. In: The Hereditary Basis of Childhood Cancer [Internet]. Cham: Springer International Publishing; 2021. p. 473–82. 10.1007/978-3-030-74448-9_16.

[19] Waldman L, Hancock K, Gallinger B, Johnstone B, Brunga L, Malkin D, et al. Perspectives and experiences of parents and adolescents who participate in a pediatric precision oncology program: “when you feel helpless, this kind of thing is very helpful.” JCO Precis Oncol. 2022. 10.1200/po.21.00444. 10.1200/PO.21.00444 35357906

[20] Bush LW, Bartoshesky LE, David KL, Wilfond B, Williams JL, Holm IA. Pediatric clinical exome/genome sequencing and the engagement process: encouraging active conversation with the older child and adolescent: points to consider - A statement of the American College of Medical Genetics and Genomics (ACMG). Genet Med. 2018;20:692–4. 10.1038/gim.2018.36. 10.1038/gim.2018.36 29565417

[21] Borry P, Evers-Kiebooms G, Cornel MC, Clarke A, Dierickx K (2009). Genetic testing in asymptomatic minors: Background considerations towards ESHG Recommendations. Eur J Hum Genet.

[22] Grootens-Wiegers P, Visser EG, Van Rossum AMC, Van Waardhuizen CN, De Wildt SN, Sweep B (2017). Perspectives of adolescents on decision making about participation in a biobank study: a pilot study. BMJ Paediatr Open.

[23] Manson NC, O’Neill O. Rethinking informed consent in bioethics [Internet]. Rethinking Informed Consent in Bioethics. Cambridge University Press; 2007. 1–212 p. 10.1017/CBO9780511814600.

[24] Savard J, Hickerton C, Metcalfe SA, Gaff C, Middleton A, Newson AJ (2020). From expectations to experiences: consumer autonomy and choice in personal genomic testing. AJOB Empir Bioeth.

[25] Lin B, Gutman T, Hanson CS, Ju A, Manera K, Butow P (2020). Communication during childhood cancer: systematic review of patient perspectives. Cancer.

[26] Byrjalsen A, Stoltze U, Wadt K, Hjalgrim LL, Gerdes AM, Schmiegelow K, et al. Pediatric cancer families’ participation in whole-genome sequencing research in Denmark: Parent perspectives. Eur J Cancer Care. 2018;1–11. 10.1111/ecc.12877. 10.1111/ecc.12877 30016002

[27] Levenseller BL, Soucier DJ, Miller VA, Harris D, Conway L, Bernhardt BA (2014). Stakeholders’ opinions on the implementation of pediatric whole exome sequencing: Implications for informed consent. J Genet Couns.

[28] Kong CC, Tarling TE, Strahlendorf C, Dittrick M, Vercauteren SM (2016). Opinions of adolescents and parents about pediatric biobanking. J Adolesc Heal.

[29] Oberg JA, Glade Bender JL, Cohn EG, Morris M, Ruiz J, Chung WK (2015). Overcoming challenges to meaningful informed consent for whole genome sequencing in pediatric cancer research. Pediatr Blood Cancer.

[30] Bertier G, Sénécal K, Borry P, Vears DF (2017). Unsolved challenges in pediatric whole-exome sequencing: A literature analysis. Crit Rev Clin Lab Sci.

[31] Grootens-Wiegers P, Hein IM, van den Broek JM, de Vries MC (2017). Medical decision-making in children and adolescents: developmental and neuroscientific aspects. BMC Pediatr.

[32] Hein IM, Troost PW, Lindeboom R, Benninga MA, Zwaan CM, Van Goudoever JB (2015). Key factors in children’s competence to consent to clinical research. BMC Med Ethics.

[33] Young MA, Thompson K, Lewin J, Holland L (2020). A framework for youth-friendly genetic counseling. J Community Genet.

[34] Taylor RM, Solanki A, Aslam N, Whelan JS, Fern LA (2014). A participatory study of teenagers and young adults views on access and participation in cancer research. Eur J Oncol Nurs.

[35] Lewis C, Hammond J, Hill M, Searle B, Hunter A, Patch C (2020). Young people’s understanding, attitudes and involvement in decision-making about genome sequencing for rare diseases: a qualitative study with participants in the UK 100, 000 Genomes Project. Eur J Med Genet.

[36] De Vries MC, Wit JM, Engberts DP, Kaspers GJL, Van Leeuwen E (2010). Pediatric oncologists’ attitudes towards involving adolescents in decision-making concerning research participation. Pediatr Blood Cancer.

[37] Mandrell BN, Johnson LM, Caples M, Gattuso J, Maciaszek JL, Mostafavi R, et al. Parental preferences surrounding timing and content of consent conversations for clinical germline genetic testing following a child’s new cancer diagnosis. JCO Precis Oncol. 2022;. 10.1200/PO.22.00323. 10.1200/PO.22.00323 PMC9848596 36265116

[38] Grootens-Wiegers P, de Vries MC, van Beusekom MM, van Dijck L, van den Broek JM (2015). Comic strips help children understand medical research. Targeting the informed consent procedure to children’s needs. Patient Educ Couns.

[39] Grootens-Wiegers P, De Vries MC, Vossen TE, Van den Broek JM (2015). Readability and visuals in medical research information forms for children and adolescents. Sci Commun.

[40] Gereis JM, Hetherington K, Robertson EG, Daly R, Donoghoe MW, Ziegler DS (2023). Parents’ and adolescents’ perspectives and understanding of information about childhood cancer precision medicine. Cancer.

[41] Johnson L, Sykes AD, Lu Z, Valdez JM, Gattuso J, Gerhardt E (2019). Speaking genomics to parents offered germline testing for cancer predisposition: Use of a 2‐visit consent model. Cancer.

[42] Mandrell BN, Gattuso JS, Pritchard M, Caples M, Howard Sharp KM, Harrison L (2021). Knowledge is power: benefits, risks, hopes, and decision-making reported by parents consenting to next-generation sequencing for children and adolescents with cancer. Semin Oncol Nurs.

[43] Alderfer MA, Zelley K, Lindell RB, Novokmet A, Mai PL, Garber JE (2015). Parent decision-making around the genetic testing of children for germline TP53 mutations. Cancer.

[44] Howard Sharp KM, Jurbergs N, Ouma A, Harrison L, Gerhardt E, Taylor L, et al. Factors associated with declining to participate in a pediatric oncology next-generation sequencing study. JCO Precis Oncol. 2020;:202–11. 10.1200/PO.19.00213. 10.1200/PO.19.00213 PMC7213582 32395682

[45] Giesbertz NAA, van Harten WH, Bredenoord AL (2019). A duty to recontact in genetics: context matters. Nat Rev Genet.

[46] Bombard Y, Brothers KB, Fitzgerald-Butt S, Garrison NA, Jamal L, James CA (2019). The responsibility to recontact research participants after reinterpretation of genetic and genomic research results. Am J Hum Genet.

[47] Knoppers BM, Sénécal K, Boisjou J, Borry P, Cornel MC, Fernandez CV (2016). Recontacting pediatric research participants for consent when they reach the age of majority. IRB Ethics Hum Res.

[48] Murad AM, Myers MF, Thompson SD, Fisher R, Antommaria AHM (2017). A qualitative study of adolescents’ understanding of biobanks and their attitudes toward participation, re-contact, and data sharing. Am J Med Genet Part A.

[49] Brothers KB, Wilfond BS. Research consent at the age of majority: preferable but not obligatory. Pediatrics. 2018;142. 10.1542/peds.2017-3038. 10.1542/peds.2017-3038 29980585

