# Supplementary material for: Experiences of pediatric cancer patients (age 12–18 years) with extensive germline sequencing for cancer predisposition: a qualitative study
Source: Eur J Hum Genet. 2024 Feb 27;32(5):567–75. doi: 10.1038/s41431-024-01565-3 (PMC11061193; doi:10.1038/s41431-024-01565-3)

Childhood cancer predisposition gene panel: PrediCT-study

The PrediCT study used a gene panel (see below) for germline testing of cancer predisposition in children with cancer. The selection criteria for this dedicated gene panel have been published before (Byrjalsen et al. 2021).

The panel will be updated regularly based on new developments in the field.The latest version of the gene panel can be found at: <https://research.prinsesmaximacentrum.nl/en/childhood-cancer-predisposition-genes>

**Reference**

Byrjalsen, Anna et al. 2021. “Selection Criteria for Assembling a Pediatric Cancer Predisposition Syndrome Gene Panel.” *Familial Cancer* 20(4): 279–87. https://link.springer.com/10.1007/s10689-021-00254-0.


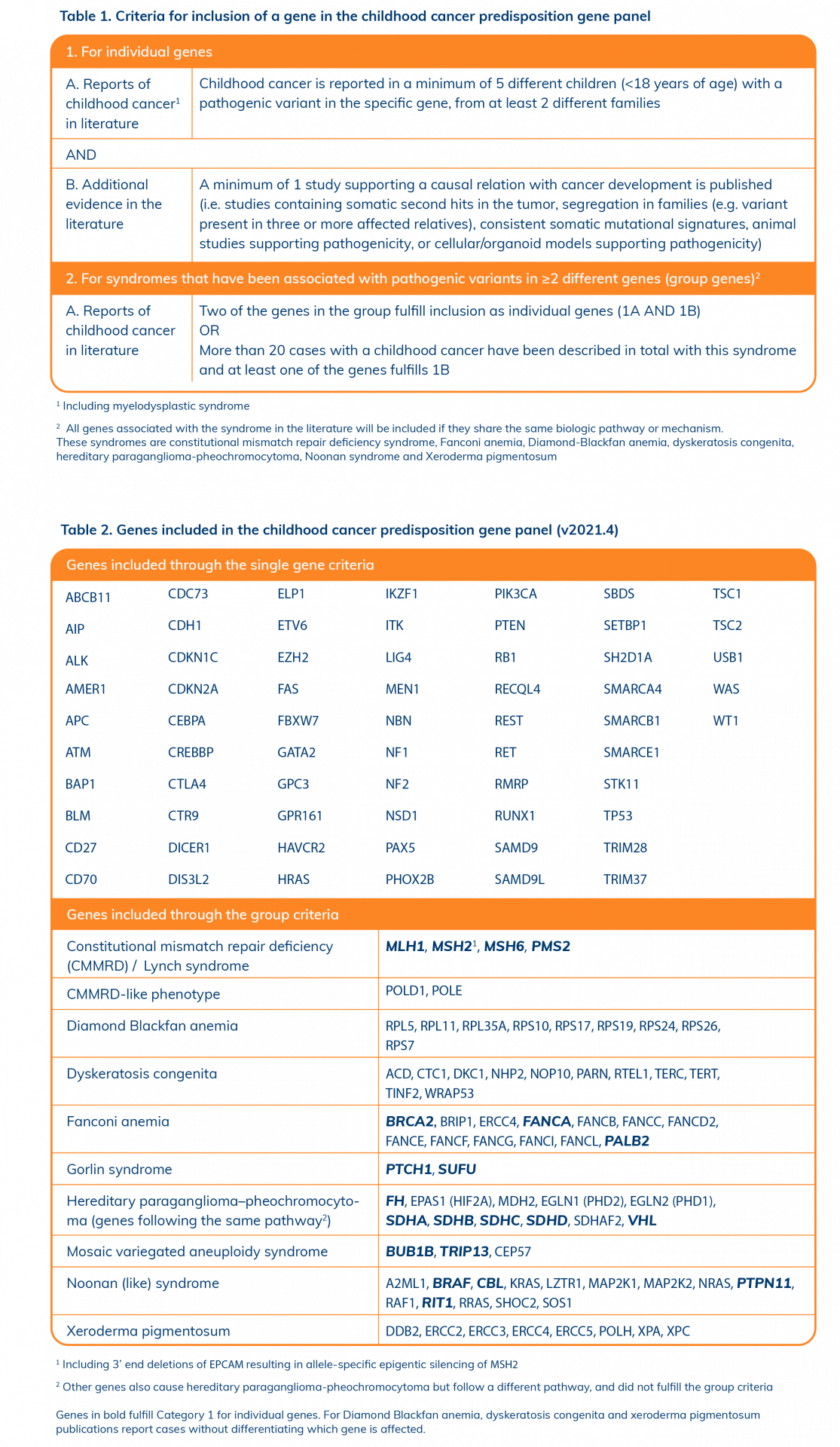

Supplement: Supplementary file 1 — Childhood cancer predisposition gene panel: PrediCT-study [file 41431_2024_1565_MOESM1_ESM.docx]
