# Supplementary material for: Experiences of pediatric cancer patients (age 12–18 years) with extensive germline sequencing for cancer predisposition: a qualitative study
Source: Eur J Hum Genet. 2024 Feb 27;32(5):567–75. doi: 10.1038/s41431-024-01565-3 (PMC11061193; doi:10.1038/s41431-024-01565-3)
Supplement: Supplementary file 2 — Interview Guide [file 41431_2024_1565_MOESM2_ESM.docx]

REFLECT-Interview guide

You have been recruited for the REFLECT interview study. We are conducting these interviews because we believe it is important to provide families with the best possible information about genetic testing. Through these interviews, we aim to learn more about your experiences so that other young people can be better informed. If there are any questions you do not want to answer or if you want to stop the interview, please say so without hesitation. As agreed, I will start the recording now.

During the interview, I will ask questions about topics discussed during the PrediCT counseling session. It is okay if you do not remember everything perfectly.

I am very curious why you want to participate in sequencing. Could you tell me more about that?

- What were your reasons for participating in the sequencing study?
- What were reasons for not participating in the sequencing study?

First spontaneous responses of adolescents were explored, if adolescents did not mention any further topics, suggestions would be introduced by the interviewer: “have you thought about *X*?”

Possible topics:

- Health benefits for yourself
- Family
- Helping future patients
- Finding out why you got ill
- Curiosity
- Impact of testing/results
- Incidental findings (predisposition for a different type of cancer)
- Future insurability

I would like to know more on how you made the decision to participate. Could you tell me about that?

## Possible questions/topics:

- Did you decide together with your parents or alone?
- How often did you discuss it with your parents?
- Did you discuss the research with others? Friends, family?
- Did you find it a difficult decision?
- Did you and your parents always agree?
- Did religion play a role in your decision?
- Did you search for additional information about the study or genetics yourself?
- What do you think about being able to participate in the decision-making or decide for yourself?
- Are you happy with how the decision-making process went?

I have brought the infographic and the information letter (from PrediCT). What did you think of the information you received?

## Possible questions/topics:

- What did you think about the information letter for children that you received?
- How would you prefer to be informed about the study?
- Do you have any suggestions for improving counseling for genetic testing in the future?

# Future considerations

## Finally, I am very curious about how you think the hospital should handle research on genetic predisposition.

## Possible questions/topics:

Re-analysis, re-consent when reaching maturity, age of consent/information.

- At what age do you think children should be involved in the decision to undergo genetic testing?
- At what age do you think children should be able to decide for themselves about genetic testing?
- Imagine that a child is too young to give consent for genetic testing. Do you think we need to contact this child when he/she reaches maturity?
- In the PrediCT study, they look at +/-140 genes known to cause childhood cancer. Imagine that in 5 years 5 new genes would be discovered, can the hospital just look at your DNA again? Do you think the hospital should ask you for permission before they do that?
